# Functional Expression of the Extracellular Calcium Sensing Receptor (CaSR) in Equine Umbilical Cord Matrix Size-Sieved Stem Cells

**DOI:** 10.1371/journal.pone.0017714

**Published:** 2011-03-17

**Authors:** Nicola Antonio Martino, Anna Lange-Consiglio, Fausto Cremonesi, Luisa Valentini, Michele Caira, Antonio Ciro Guaricci, Barbara Ambruosi, Raffaele Luigi Sciorsci, Giovanni Michele Lacalandra, Stephan Joel Reshkin, Maria Elena Dell'Aquila

**Affiliations:** 1 Department of Animal Production, Faculty of Biotechnological Sciences, University of Bari, Valenzano, Bari, Italy; 2 Reproduction Unit, Large Animal Hospital, Faculty of Veterinary Medicine, University of Milan, Lodi, Italy; 3 Department of General and Environmental Physiology, Faculty of Science, University of Bari, Bari, Italy; Centro Cardiologico Monzino, Italy

## Abstract

**Background:**

The present study investigates the effects of high external calcium concentration ([Ca^2+^]_o_) and the calcimimetic NPS R-467, a known calcium-sensing receptor (CaSR) agonist, on growth/proliferation of two equine size-sieved umbilical cord matrix mesenchymal stem cell (eUCM-MSC) lines. The involvement of CaSR on observed cell response was analyzed at both the mRNA and protein level.

**Methodology/Principal Findings:**

A large (>8 µm in diameter) and a small (<8 µm) cell line were cultured in medium containing: 1) low [Ca^2+^]_o_ (0.37 mM); 2) high [Ca^2+^]_o_ (2.87 mM); 3) NPS R-467 (3 µM) in presence of high [Ca^2+^]_o_ and 4) the CaSR antagonist NPS 2390 (10 µM for 30 min.) followed by incubation in presence of NPS R-467 in medium with high [Ca^2+^]_o_. Growth/proliferation rates were compared between groups. In large cells, the addition of NPS R-467 significantly increased cell growth whereas increasing [Ca^2+^]_o_ was not effective in this cell line. In small cells, both higher [Ca^2+^]_o_ and NPS R-467 increased cell growth. In both cell lines, preincubation with the CaSR antagonist NPS 2390 significantly inhibited the agonistic effect of NPS R-467. In both cell lines, increased [Ca^2+^]_o_ and/or NPS R-467 reduced doubling time values.Treatment with NPS R-467 down-regulated CaSR mRNA expression in both cell lines. In large cells, NPS R-467 reduced CaSR labeling in the cytosol and increased it at cortical level.

**Conclusions/Significance:**

In conclusion, calcium and the calcimimetic NPS R-467 reduce CaSR mRNA expression and stimulate cell growth/proliferation in eUCM-MSC. Their use as components of media for eUCM-MSC culture could be beneficial to obtain enough cells for down-stream purposes.

## Introduction

Mesenchymal stem cells (MSCs) are undifferentiated cells able to self-renew, that have a high proliferative capacity and grow as adherent cells [1]. Several reports suggest that MSCs are able to differentiate into various cell types, including chondrocytes, osteocytes, adipocytes, myocytes, cardiomyocytes and neurons [2], [3]. MSCs can be isolated from different adult tissues, such as bone marrow (BM), adipose tissue, dental pulp; in extraembryonic tissues, such as placenta and umbilical cord (UC) and from a variety of fetal tissues, such as spleen, lung, pancreas, liver, kidneys, amniotic fluid [1], [4]–[6]. Recently, several groups reported success, both in humans and in large animal models, in isolating and establishing MSCs cultures from fetal adnexa such as amniotic membrane, amniotic fluid or UC (human:[7], [8], pigs [9], horses [10]–[15] and dogs [16]–[18]. The process through which these cells can be obtained is non-invasive, painless and without harm for the mother or the infant [1], [10], [11].

Several studies have been performed to determine the possibility to obtain MSCs from UC matrix (UCM) or stroma, namely Wharton's jelly, the primitive connective tissue of UC [1], [5], [8]. Wharton's jelly in UC surrounds a set of two arteries and one vein and is composed by a mucous connective tissue rich in proteoglycans, mainly hyaluronic acid, and specialized fibroblast-like cells [3]. By using Wharton's jelly, a significantly greater number of MSCs can be isolated than in other adult or fetal tissue such as BM or UC blood [7]. MSCs have been isolated from three regions of Wharton's jelly: the perivascular zone, the intervascular zone and the subamnion. At the present time, it is unknown whether MSCs isolated from these different compartments could represent different cell populations [5]. Wharton's jelly MSCs do not present an univocal phenotype, but they show markers that are present on other cellular lineages. Immunophenotyping by flow cytometry revealed that these cells are positive for specific MSC markers such as CD73, CD90, CD105 [3], [19]–[21] and they are negative for the hematopoietic line markers such as CD34 and CD45 [1], [3], [19]–[22]. These cells are also negative for the human leukocyte antigen HLA-DR [1], [3], [19], [20], [22], [23] thus suggesting a potential role as a human allogenic cell source for cell-based therapies. Many works have reported that Wharton's jelly MSCs can be induced to differentiate in vitro into adipogenic, osteogenic and chondrogenic lineages [1], [3], [8], [19]–[22], [24]–[26], cardiomyocyte [3], [8], [24], [27] and neural-like cells [3], [8], [20], [22], [28], [29].

In the past few years, attention has been drawn to MSCs because of their potential use in cell therapy and regenerative medicine in several clinical fields. In parallel with advancing of studies on MSC, the urgency of establishing suitable animal models that could allow researchers to study the properties of MSCs and their use in different pathological conditions has become more evident. Stem cell and tissue engineering research in the horse has exciting comparative and equine-specific perspectives that most likely will benefit the health of horses and humans [30]. Recently, first reports on the possibility to isolate, to characterize and to grow *in vitro* equine umbilical cord matrix mesenchymal stem cells (eUCM-MSCs) have appeared in the literature [10]–[13], [31]. Equine UCM is a source of primitive, mesenchymal stem cells that can be cultured, cryogenically preserved and differentiated in adipocytes, chondrocytes, osteoblasts and in cells with a morphology typical of neurons with axon- and dendrite-like processes [10], [11]. These cells showed expression of the embryonic markers Oct-4, SSEA-4 and c-Myc that is a regulator of early gene expression and cell cycle progression in a variety of proliferating cells. Equine UCM-MSCs (eUCM-MSCs) also expressed a number of antigens associated with pluripotent adult stem cells, including CD54, CD90, CD105, and CD146. There was no significant expression of HLA-ABC, HLA1AG, and MHC II [10]. Cremonesi et al., 2008; [12] showed that eUCM-MSCs express mRNA for Oct-4 and Sox-2 markers.

Extracellular calcium (Ca^2+^) is a potent mediator of the balance between proliferation and differentiation in a number of different cell types [32], [33]. Expression of the G protein-coupled calcium-sensing receptor (CaSR) has been demonstrated in Ca^2+^ responsive epithelial and mesenchymal cell types, thus suggesting a possible mechanism by which Ca^2+^ could induce changes in proliferation [33]–[36].The CaSR plays a key role in the regulation of whole-body Ca^2+^ metabolism [32], [37], [38]. The molecular identification of CaSR in bovine parathyroid cells by Brown et al., 1993; [39] opened up the possibility that Ca^2+^ can be considered as a first messenger outside cells [39], [40]. The CaSR was originally named for its ability to detect/transduce subtle but physiologically meaningful changes in extracellular Ca^2+^ concentration ([Ca^2+^]_o_). However, it responds to many other bivalent and trivalent cations, to changes in other physiological parameters such as L-amino acids, polyamines, ionic strength, pH, and to drugs such as aminoglycosidic antibiotics, calcimimetics, and calcilytics [32], [41]. CaSR-null mice exhibit loss of feedback control of parathyroid hormone secretion, hyperparathyroidism, and metabolic bone diseases. Moreover, inactivating and activating mutations of the receptor in humans have been shown to induce various disorders of Ca^2+^ metabolism [41]. CaSR has been reported to be widely expressed in several mammalian tissues, including tissues that are not clearly involved in Ca^2+^ metabolism such as brain, lens epithelial cells, pituitary gland, bone marrow, peripheral blood, breast ductal cells, pancreas, keratinocytes, ovarian surface epithelial cells, parathyroid gland stem cells and the gastrointestinal system [32], [33], [39], [41]–[43]. CaSR localization in cells that seem to have no functional relationship with the maintenance of the whole-body Ca^2+^ balance has been linked to the regulation of different cellular processes, such as secretion, chemotaxis, apoptosis, cell proliferation, differentiation, and ion channel activity [2], [41].

The aim of the present study was to evaluate the effects of [Ca^2+^]_o_ and a calcimimetic, NPS R-467 known as a selective CaSR agonist, on growth/proliferation of two equine size-sieved UCM-MSC lines. The involvement of the extracellular CaSR on observed stimulation of growth/proliferation in these cell lines was investigated by evaluating added compounds on CaSR mRNA and protein expression.

## Results

### Experiment 1: Effects of high external calcium concentration and the calcimimetic NPS R-467 on growth/proliferation of equine size-sieved UCM-MSCs

**Figure 1:**
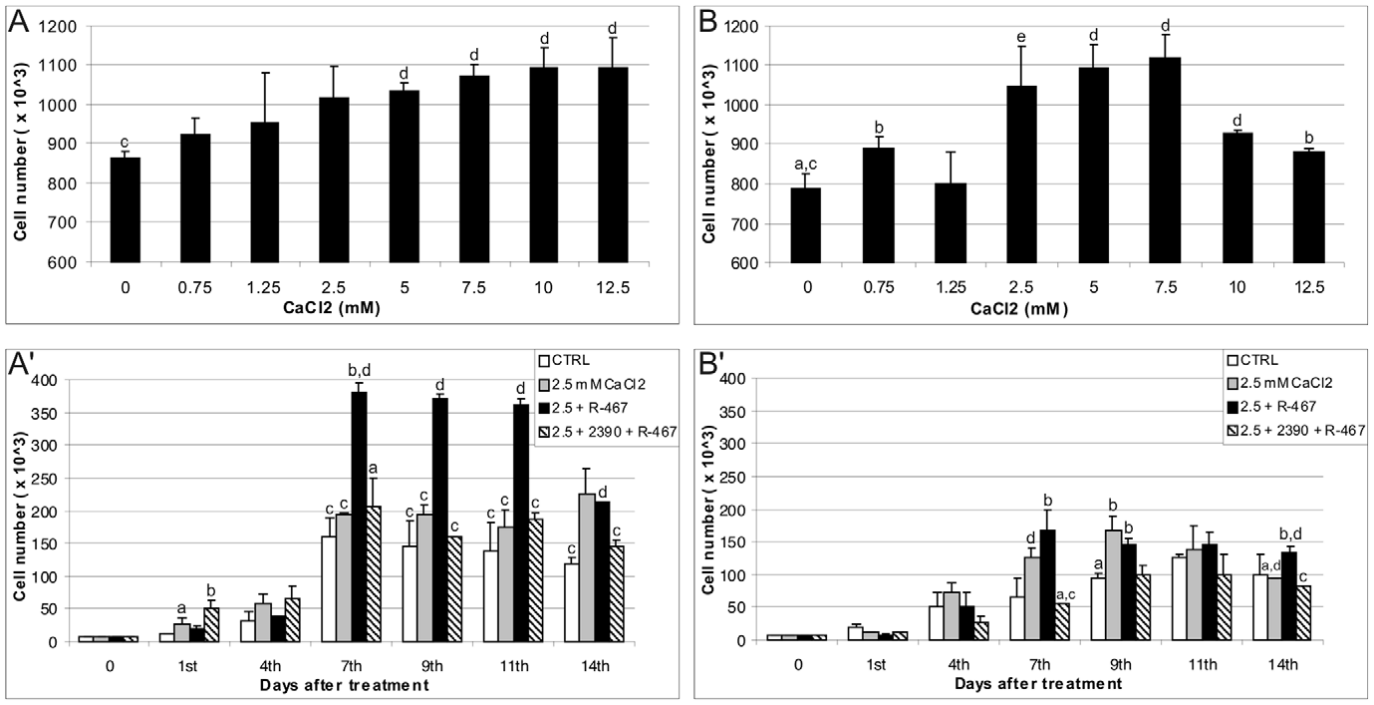
Effects of Ca^2+^, the calcimimetic NPS R-467 and the CaSR antagonist NPS2390 on eUCM-MSC growth. Cells were plated in 6-wells plates and treated as described in materials and methods. Effects of increasing doses of extracellular Ca^2+^ on both, large (A) and small (B), cell lines. In the large cell line (A'), increasing Ca^2+^ concentration to 2.5 mM was not effective on cell growth. In the presence of 2.5 mM Ca^2+^, the allosteric activator of the CaSR, NPS R-467 (3 µM) significantly increased the cell number/well from day 7^th^ after treatment (P<0.001). In the small cell line (B'), increasing Ca^2+^ concentration induced cell growth on day 9^th^ (P<0.05). In the presence of 2.5 mM Ca^2+^, the allosteric activator of the CaSR, NPS R-467 significantly increased the cell number/well on day 9^th^ (P<0.05). In both cell lines, 30 min preincubation in presence of the CaSR antagonist NPS 2390 (10 µM) significantly inhibited the agonist effect. Results are mean ± SD of 2 data points. Student's t Test: a vs b: P<0.05; a vs e: P<0.01; c vs d P<0.001.

Increasing external Ca^2+^ to 2.5 mM did not affect cell growth, in the large (Fig. 1A') cell line, whereas in the small cell line (Fig. 1B'), it significantly increased cell growth compared with controls (P<0.05). The addition of NPS R-467, in presence of 2.5 mM exsternal Ca^2+^, in both cell lines significantly increased cell growth compared with controls (P<0.001 for both the large cell line and P<0.05 for the small cell line). In detail, in the growth study, the large cell line was not stimulated by increased Ca^2+^ but it was stimulated by NPS R-467 both earlier (from day 7) and strongly (P<0.001) (Fig. 1A'). This may lead to the hypothesis that Ca^2+^, in this cell line, could have primed the receptor to the effects of its selective agonist NPS R-467. On the other hand, the small cell line responded both later (on day 9) and more weakly (P<0.05) stimulated by both Ca^2+^ and calcimimetic NPS R-467 (Fig. 1B'). In both cell lines, preincubation with the CaSR antagonist NPS 2390 inhibited the agonistic effect of NPS R-467.

Additionally, DT evaluation in all examined culture conditions were performed with the aim to evaluate long term effects of tested compounds on the eUCM-MSC proliferation rate. Interestingly, it was observed that, in both cell lines, cell proliferation rate increased (approximately 3 fold) whenever extracellular Ca^2+^ was increased (2.5 mM, 2.5 mM+R-467 and 2.5 mM+2390+R-467). It can be hypothesized that elevating [Ca^2+^]_o_ in culture medium is beneficial to increase UCM-MSC proliferation rate at later passages. DT values were calculated from P5 to P8 for both, large and small, cell populations. The stimulatory effects of additional Ca^2+^ and NPS R-467 on cell proliferation occurred at P8 in the large cell line and from P7 in the small cell line. Whenever additional Ca^2+^ was added, reduced cell DTs were observed compared with controls (large cell line, DT values = 5.59, 4.03 and 5.59 for 2.5 mM CaCl_2_, 2.5 mM+NPS R-467 and 2.5 mM+NPS 2390+NPS R-467 respectively vs 12 in CTRL; small cell line, DT values = 4.03, 5.59 and 2.90 respectively vs 12 in CTRL), thus evidencing a stimulatory effect of increasing Ca^2+^ concentration on cell proliferation of UCM-MSCs.

### Experiment 2a: Effects of higher external calcium and the calcimimetic NPS R-467 on the relative abundance of the CaSR transcript in equine UCM-MSCs

**Figure 2:**
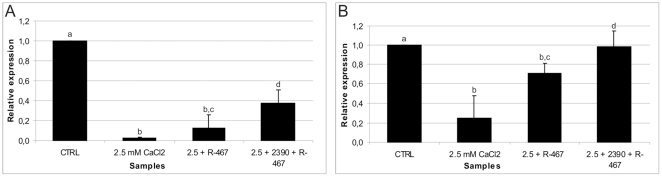
CaSR transcript relative abundance in eUCM-MSCs upon Ca^2+^- or calcimimetic-induced CaSR stimulation. Quantitative Real Time RT-PCR analysis of the CaSR transcript in Ca^2+^ (2.5 mM CaCl_2_) and calcimimetic-treated (2.5+R-467) equine UCM-MSCs versus controls (CTRL) or cells pre-incubated with the CaSR antagonist (2.5+2390+R-467). Cells were examined on day 4^th^ after treatments. For each sample, data (mean ± SD of three independent experiments in duplicate, CaSR average Ct) were normalized relatively to the abundance of HPRT1 mRNA (endogenous control) and normalized values were compared among groups. In the large cell line (panel A), CaSR transcription was strongly down-regulated in presence of additional Ca^2+^ (2% of control value; vs b: P<0.001) and NPS R-467 (13%; a vs b: P<0.001). In the small cell line (panel B), CaSR transcription was again down-regulated by additional Ca^2+^ (25%; a vs b: P<0.001) and NPS R-467 (72%; a vs b: P<0.001) even at a lesser extent. In both cell lines, the CaSR antagonist NPS 2390 reversed the effects of NPS R-467 (38% and 97%, for the large and the small cell line, respectively; c vs d: P<0.05).

In the large cell line, the CaSR transcript level decreased in higher Ca^2+^- and agonist-treated cells compared with controls. Its relative abundance was reduced to 2% of the control value in higher Ca^2+^-(P<0.001) and to 13% in agonist-treated cells (P<0.001). In antagonist-pretreated cells, the CaSR transcript level increased up to approximately 3 fold compared with agonist-treated cells (0.38 *vs* 0.13; P<0.05, Fig. 2A).

In the small cell line, in higher Ca^2+^-treated cells, the CaSR transcript level was approximately a quarter of the value observed in control samples (P<0.001). In this cell line, the relative abundance of the CaSR transcript was reduced to 72% of control levels upon agonist treatment (P<0.001; Fig. 2B). In cells pre-incubated in the presence of the CaSR antagonist NPS 2390, higher amounts of the CaSR transcript were found (0.97) compared with those found in agonist-treated cells (0.72; P<0.05). The relative abundance in antagonist pre-treated cells was similar to that of controls (reference sample value = 1; Fig. 2B).

### Experiment 2b: Effects of increased external calcium concentration and the calcimimetic NPS R-467 on CaSR protein expression and subcellular localization in equine UCM-MSCs

**Figure 3:**
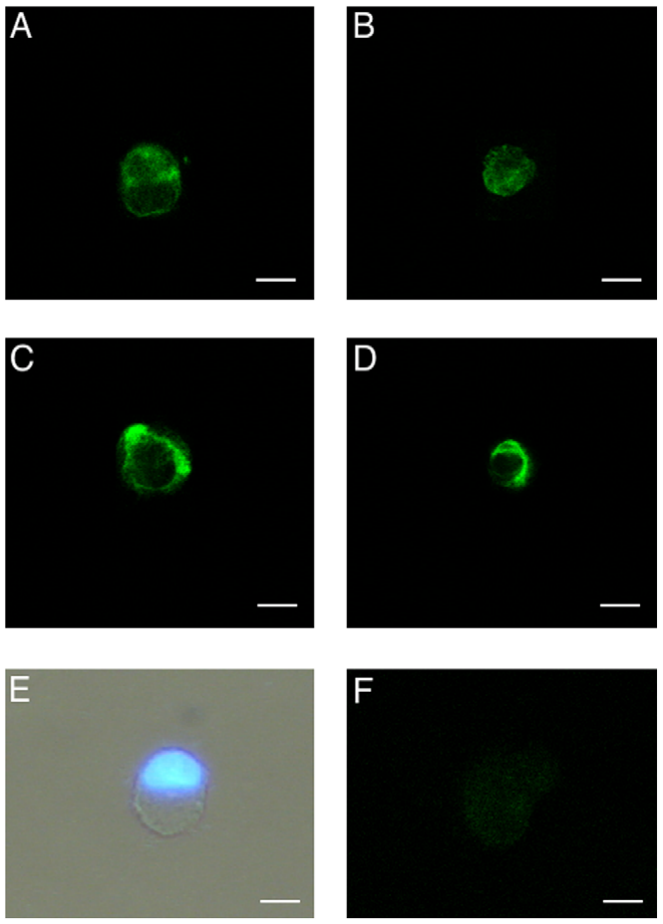
CaSR protein expression and subcellular localization in eUCM-MSCs upon Ca^2+^- or calcimimetic-induced CaSR stimulation. Detection of CaSR expression in equine UCM-MSCs in the >8 µm cell line (A, C) and <8 µm cell line (B, D) by immunofluorescence with a primary antibody against a 20 amino acid peptide sequence near the C-terminus of human CaSR and observation by confocal laser scanning microscopy. In both cell lines, cells showing CaSR labeling either predominantly evident whitin the cytoplasm (A, B) or on the plasma membrane (C, D) were present. For each cell, scanning was conducted with 12 optical series from the top to the botton of the cell with a step size of 0.45 µm and images were taken to the equatorial plane. Representative photomicrograph of equine UCM-MSC as observed after thawing, staining with Hoechst 33258 and observed under phase contrast microscopy merged with UV light epifluorescence (E). In this cell, regular round shape morphology and an eccentric nucleus can be seen. Negative minus primary control (F). Scale bar represent 20 µm (A, C, E, F) or 10 µm (B, D).

The CaSR protein was found to be expressed (green labeling) in eUCM-MSCs of both cell lines cultured in all examined conditions. In all samples, marked staining in light green was seen with an ubiquitous distribution within the cytoplasm and along the plasma membrane. Interestingly, in the large cell line, treatments with higher Ca^2+^ or higher Ca^2+^ plus the CaSR agonist increased the number of cells showing marked labeling on the plasma membrane and reduced the rate of cells showing prevailing CaSR labeling in the cytosol, compared with controls. In detail, after treatment with higher Ca^2+^, the difference approached statistical significance (18/44, 41% vs 8/40, 20%; Chi-square  = 3.36) whereas it was statistically significant (20/40, 50% vs 8/40, 20%; P<0.05) in cells cultured in the presence of both Ca^2+^ and CaSR agonist (Fig. 3A–D). In the small cell line, neither increased Ca^2+^ concentration nor the addition of CaSR agonist addition had any effect on CaSR protein localization. Cells showed a round shape and a vescicular large nucleus (Fig. 3E). No staining was detected in negative controls (minus primary antibody controls; Fig. 3F).

## Discussion

### Kinetic study

After preliminary testing of CaSR expression in eUCM-MSCs of both cell lines by immunocytochemistry, the first purpose of this study was to determine whether CaSR activation, by means of increased Ca^2+^ or calcimimetic NPS R-467 addition, could stimulate growth/proliferation of eUCM-MSCs. In both cell lines, cell growth/proliferation was stimulated by Ca^2+^ or calcimimetic NPS R-467 addition and their effects were reversed by the CaSR antagonist NPS 2390 even if the two cell lines behaved differently.

Our data are in agreement with previous studies where several cell systems have been shown to respond differently to Ca^2+^ or NPS R-467 treatments in relation to their effects on cell proliferation which can be stimulated or inhibited to a different extent [32], [44]–[46].

The calcimimetic NPS R-467 is a phenylalkylamine compound, able to potentiate the effects of extracellular Ca^2+^ on CaSR [48]–[52] and has been reported to act through the transmembrane domain of CaSR by increasing the affinity of the receptor for its cognate G proteins or by enhancing the signal transduction from the ‘head’ of the CaSR to its intracellular domains [53]–[55]. Here, in both examined cell lines we observed that the allosteric activator of the CaSR NPS R-467 increased cell growth and this effect was inhibited by adding the CaSR antagonist NPS 2390. These data provide evidence that the NPS R-467-induced cell growth is a CaSR-mediated response. Our observations are in agreement with previous studies in which it was reported that NPS R-467 and the NPS R-467 precursor, NPS R-568, stimulate cell mitosis [33], [35], [44], [56]–[58] and oocyte meiosis [59].

As a confirmation to the observed CaSR-mediated effect on eUCM-MSC, we tried to revert the stimulatory effect observed in the presence of NPS R-467 by using a CaSR antagonist, NPS 2390. As previously observed, NPS 2390 is a potent and selective non-competitive group I metabotropic glutamate receptor (mGluR) antagonist [60]. In relation to CaSR's reportedly high structural homology with mGluR1 [52], NPS 2390 has been used as CaSR antagonist in previous studies [61], [62]. Indeed, in the present study, the effect of NPS R-467 was significantly reduced by pre-incubation in the presence of NPS 2390 in both cell lines even at different time points (from day 7 for the large cell line and on day 7 and 14 in the small cell line). These data are in agreement with previous studies reporting effects of NPS 2390 in reducing intracellular Ca^2+^ concentration [61], [62], cell proliferation [62] and the rate of oocytes reaching meiotic maturation [59].

Taken together, our data indicated that CaSR activation may lead to an increase of growth/proliferation rate in eUCM-MSCs. However, different responses were obtained in the two examined cell lines upon treatment with increased [Ca^2+^]_o_ or the CaSR agonist NPS R-467. The different response to treatments between the two examined cell lines suggested that CaSR could be differentially expressed and activated in these cell lines.

### Effects on CaSR expression

In order to confirm that both the Ca^2+^ and the calcimimetic NPS R-467- induced cell proliferation in eUCM-MSCs were CaSR-mediated responses, the second group of experiments were aimed at testing whether CaSR expression was modified, at mRNA and/or protein level, upon treatments. In order to evaluate modifications preceding cell proliferative responses, samples were examined on day 4 after treatments. Detection of CaSR expression was confirmed, both at mRNA and at protein level, in both cell lines.

In the large cell line (strongly stimulated to proliferate upon agonist treatment), CaSR transcription was strongly down-regulated both upon Ca^2+^ and calcimimetic plus agonist treatment. In the small cell line (weakly stimulated to proliferate upon Ca^2+^ and agonist treatment), CaSR transcription was significantly down-regulated by Ca^2+^ and CaSR agonist treatment even if at lesser extend. At the present time, these findings could be only interpreted as being related to hypotheses reported in previous studies. As an example, Roussanne et al. 2001; [65] reported that an increase in [Ca^2+^]_o_ stimulates, while the calcimimetic NPS R-467 inhibits, the *in vitro* proliferation rate of parathyroid cells derived from uraemic patients, known to express reduced CaSR levels. In the attempt to explain these apparent discrepancies those authors hypothesized that calcium may regulate cell proliferation by two different pathways: 1) with predominant growth inhibition in cases of high CaSR expression or activation, but with prevailing stimulation of proliferation in cases of low CaSR expression.

In the present study, extracellular Ca^2+^ and the calcimimetic NPS R-467 regulated cell proliferation with strong stimulation in the case of strongly lowered CaSR relative expression (as observed in the large cell line) but weak stimulation in the case of weakly lowered relative CaSR abundance (as observed in the small cell line). There are a relatively few studies available to date on the relation between CaSR mRNA/protein relative abundance and cell proliferation. However, most of in vitro studies yielded different results: to our knowledge, three studies reported increased CaSR expression upon high Ca^2+^ in vitro exposure [66]–[68]; two other studies found no effect [69]–[70].

A possible explanation of this cell context-dependent response might be found in the reports that the CaSR gene has promoter regions sensitive to both Ca^2+^ and Vitamin D. Response elements for the two regions could determine what the answer whether an “up” or a “down-regulation” of CaSR expression [66]. It could be hypothesized that in a stem cell population, not yet committed to undergo differentiation (maintained in culture in the absence of differentiation stimuli), extracellular Ca^2+^ elevation could either induce an interaction of the promoter with some elememt specific to the type of stem cell through a different promoter structure or, through a still unknown post-translational mechanism of regulation of CaSR by Ca^2+^ or its mimicks, produce a CaSR down-regulation and cell growth/proliferation. Further studies are necessary to fully explore the mechanism of Ca^2+^-induced CaSR expression in UCM-MSCs.

In addition, CaSR mRNA, in eUCM-MSCs issuing from the two cell lines examined in the present study, may be 1) regulated by different promoters, as reported in other cell systems [64], 2) be present in two different allelic forms to produce two different protein isoforms that can be both detected by the antibody used in the present study; 3) have different signal transduction pathways/mechanisms that may differentially regulate the response to CaSR stimulation. These hypotheses could be related to observations by Roussanne et al., 2001; [65]. Again, the occurrence of post-translational modifications of the receptor (such as glycosylation, phosphorylation or ubiquitination, etc) during the process of differentiation/growth of these two cell lines or the presence of different protein amounts could be hypothesized.

CaSR protein in eUCM-MSCs was identified and localized within the cytoplasm and in the cortical region. As previously observed in other cell systems, the receptor present on the cell surface could represent the mature form of the receptor whereas the cytoplasmic CaSR could simply represent the nascent receptor protein, undergoing post-translational modifications, even if it cannot be excluded that it could have a distinct biological function, such as intracellular Ca^2+^ sensing [71], [72]. This dual CaSR staining pattern, cytosolic and on the cell surface, has been observed in previous studies performed in osteoblastic cell lines (human, [73]; rat, [63]) as well as in rat oligodendrocytes [69], rat microglia [57], [63], mouse mesangial cells [62], human and equine cumulus-oocyte complexes and ovarian granulosa cells [74], [59]. In the large cell line treated with Ca^2+^ or with Ca^2+^ plus NPS R-467, CaSR immunostaining increased at cortical level and was reduced in the cytosol. The difference approached statistical significance in cells treated with calcium and was statistically significant (P<0.05) in cells treated with Ca^2+^ plus NPS R-467. This finding further supports our hypothesis that these compounds activate CaSR in eUCM-MSCs leading to cell CaSR-mediated cell proliferation. Our data are in agreement with data obtained in a previous study from our unit in equine cumulus and granulosa cells [59].

### Conclusions

In conclusion, we show that CaSR is expressed at mRNA and protein levels in eUCM-MSCs, and is functionally active since calcium and the selective CaSR agonist NPS R-467 induced a stimulatory effect on cell growth/proliferation and this effect was reversed by pre-incubation with the CaSR antagonist NPS 2390. The CaSR agonist improved cell growth/proliferation in the presence of physiological [Ca^2+^]_o_. In the two examined cell lines, the CaSR transcript was strongly (large) or weakly (small) down-regulated on day 4 after treatment, leading respectively to strong or weak increase in cell growth/proliferation. In cells showing the stronger proliferative response (large cell line), treated with Ca^2+^ plus NPS R-467, CaSR immunostaining increased at membrane level and was reduced in the cytosol. Taken together, these data provide a significant contribution to the knowledge of CaSR expression and regulation mechanism in UCM-MSC from UC. Observed differences between analyzed cell lines could be related to differences in their developmental and functional stage, as reported by Corradetti et al., 2010 [13] who suggested that in the perivascular portion of UCM, the large cells are mature MSC and the smaller ones are recycling stem cells. Increasing calcium concentration and adding NPS R-467 in culture media for eUCM-MSC might be beneficial in order to obtain a desirable result of increasing the cell number and reducing the time interval necessary to obtain enough cells for downstream therapeutic purposes.

## Materials and Methods

### Chemicals

NPS R-467 ((R)-N-(3-phenylpropyl)-a-methyl-3-methoxybenzylamide hydrochloride was a kind gift of Dr. R. Caroppo, Department of General and Environmental Physiology, Faculty of Biological Sciences, Via Orabona Bari Italy). NPS 2390 (Quinoxaline-2-carboxilic acid adamantan-1-ylamide) was purchased by Sigma (N 4786), Milan Italy.

### Cell lines

The study was carried out on two homogeneous subpopulations of eUCM-MSCs both isolated from perivascular Wharton's jelly by using a particular culture device: multi-dishes with transwell inserts of 8 µm pores as described by Corradetti et al., 2010; [13]. Therefore, we have referred to the cells as size-sieved stem cells separated by their dimension. In this way, two types of adherent cells appeared after seeding, one larger than 8 µm (namely, large cell line) and the other smaller than 8 µm in diameter (namely, small cell line). These two cell lines were previously reported as positive for MSC markers (CD105, CD44, CD29) and negative for the emopoietic CD34 marker as assessed by RT-PCR [13].

### Culture conditions

Cells were cultured at 38.5°C in a humidified atmosphere (95%) under 5% CO_2_ in standard expansion medium consisting of Dulbecco's Modified Eagle's Medium - High Glucose (DMEM) (Sigma D-5546) supplemented with 10% Fetal Calf Serum (FCS) (Sigma F3018), 100 U/ml penicillin, 100 µg/ml streptomycin, 0.25 µg/ml amphoterycin solution (Sigma A-5955), 2 mM L-glutamine (Sigma G-7513) and 10 ng/ml Epidermal Growth Factor (EGF; Sigma E-9644).

### Cell freezing

After trypsin treatment and count, cells of each well were resuspended in standard medium supplemented with 10% (v/v) FCS and 10% (v/v) dimethyl sulfoxide (DMSO, Sigma D-5879) and cryopreserved in cryotubes that were stored at −80°C and then transferred to cryogenic containers with liquid nitrogen until molecular and immunofluorescence analysis.

### Reverse Transcription PCR

The Cell-to-cDNA II kit (AM1722, Ambion, Monza, Milan, Italy) was used to produce cDNA. After thawing, cells were washed in PBS and then heated in Cell Lysis II buffer at 75°C for 10′ to release RNA after rupture of the cells and to inactivate RNases. Next, the cell lysate was treated with DNase I to degrade genomic DNA (37°C for 15′) and after that, DNase was inactivated by heating to 75°C for 5′. Five to 10 µL cell lysate was added with 4 µL dNTP Mix, 2 µL of random decamers and carried to 16 µL with nuclease-free water then was heated 3 min at ∼70°C. Next, the remaining RT reagents were added: 2 µL 10X RT Buffer, 1 µL M-MLV Reverse Transcriptase, 1 µL RNase Inhibitor, mixed gently and centrifuged briefly. For the minus-RT control, water was added at this time point. Reaction tubes were incubated at 42°C for 15–60 min, then at 92–95°C for 10 min to inactivate the reverse transcriptase.

### Real Time PCR

Real Time PCR was performed by using Real Time TaqMan technology and analyzed on automated “StepOne System” (Applied Biosystem, Monza, Milan Italy). The CaSR primers and probe were designed, intron-spanning, using Primer Express 3.0 (Applied Biosystem) on the basis of equine CaSR mRNA (GenBank accession no. GI255653075) and were as follows: CaSR Real Time PCR primers: forward primer: 5′-CTTGGCAGGTCCTGAAGCA-3′; reverse primer: 5′-TGGTTGTTATACCCCCTGGTC-3′; CaSR hybridization probe: 5′-CTACGGCACCTCAAC-3′ (Applied Biosystem). The CaSR hybridization probe, which binds to PCR products, was labeled with a reporter dye (6-carboxy-fluorescein, FAM) on the 5′ nucleotide and a quenching dye (MGB, minor groove-binder) with NFQ (non fluorescent quencher) on the 3′ nucleotide where MGB hyper-stabilized duplexes with complementary DNA. TaqMan equine Hypoxanthine phosphoribosyltransferase 1 (HPRT1), inventoried by Applied Biosystem (EC03470220_M1), was used as endogenous control. Samples were run in duplicates on Microamp fast optical 48-well reaction plate (Applied Biosystem) where twenty-microliters reactions for each well contained: 10 µL TaqMan gene expression Master Mix 2X (Applied Biosystem), 1 µL 900 nM Primers, 250 mM Probe, 4 µL cDNA, 5 µL: RNase Free H_2_O. Cycling parameters were: 2 min at 50°C, 10 min at 95°C followed by 47 cycles of 15 s at 95°C and 1 min at 60°C. Data were collected by using the OneStep Software and relative quantification was performed by using comparative method after determining the Ct (threshold cycle) values for the reference (HPRT1) and the target gene (CaSR) in each sample sets, according to the 2^-ΔΔCt^ method as described by the manufacturer. Changes in mRNA expression levels were calculated after normalization to HPRT1. The program calculates the ΔCt and the ΔΔCt with the formulas below: ΔCt  =  Ct_Mean(HPRT1) - Ct_Mean(CaSR); ΔΔCt  =  ΔCt - ΔCt_Mean, so that the gene expression level = 2^−ΔΔCt^. Changes in gene expression were reported as percentage changes relative to controls.

### Immunofluorescence

After collection by trypsin digestion, cells of each treatment (as reported in the Experimental design, see below) were analyzed for CaSR protein expression as previously reported by Dell'Aquila et al., 2006; [69] and De Santis et al., 2009; [59] for ovarian cumulus and granulosa cells. Briefly, cells were seeded on polylisinated slides and fixed in Aceton (Sigma 20083-044) for 1 h, washed in 100 mM glycine in Phosphate Buffered Saline (PBS), and incubated for 30 min in 1% (w/v) Bovine Serum Albumin (BSA) in PBS (PBS-BSA). Cells were then incubated overnight at room temperature in a 1∶2500 dilution of a primary rabbit polyclonal antibody against a 20 amino acid peptide sequence near the C-terminus of human CaSR (antiCaSR C0117-15 US Biological, Swampscott, MA, USA). Cells incubated overnight in PBS-BSA were used as negative controls (minus primary controls). At the end of incubation, cells were washed in PBS, then incubated for 2 h at room temperature with a fluorescein isothiocyanate (FITC)-conjugated rabbit IgG-secondary antibody, diluted 1∶200 in PBS and evaluated by confocal microscopy as described below. Evaluation of cell morphology, by phase contrast microscopy, and nuclear chromatin, by epifluorescence microscopy after 2.5 µM Hoechst 33258 (Sigma B-1155) staining (in 3/1 glycerol/PBS solution), were also performed.

### Confocal laser scanning microscopy and image analysis

Cells were observed at 600×magnification in oil immersion with a laser-scanning confocal microscope (C1/TE2000-U Nikon) equipped with the Argon Ions 488 laser and the 495–519 (B2-A) nm excitation/emission filter. For each field, scanning was conducted with 12 optical series from the top to the bottom of the cell with a step size of 0.45 µm. Parameters related to fluorescence intensity (x63 objective, zoom  = 0, 30 µm pinhole size) were maintained at constant values for all measurements.

### Experimental design

#### Experiment 1: Effects of high external calcium concentration and the calcimimetic NPS R-467 on growth/proliferation of equine size-sieved UCM-MSCs

At a preliminarily level, a dose-response study of the effects of extracellular Ca^2+^ on UCM-MSCs was performed in order to identify the best concentration of Ca^2+^ in subsequent NPS R-467 experiments. The large and small cells were seeded in six well plates and cultured in the standard medium supplemented with rising [Ca^2+^]_o_ from 0 to 12.5 mM CaCl_2_. Cell counts were performed on days 1^st^ and 4^th^ after treatments. Calcium induced cell growth in both large and small UCM-MSC lines in a dose-dependent manner. This effect was observed at day 4^th^ (Fig 1 A,B) after treatment whereas no effect was found at day 1^st^ (data not shown). Given that in both cell lines the rising phase was comprised between 0 and 5 mM CaCl_2_ and the maximal response was observed at 5, 7.5 and 10 mM, subsequent experiments were performed at 2.5 mM CaCl_2_ which was considered as EC_50_. External calcium concentration in the present study was actually either 0.37 or 2.87 mM Ca^2+^. Infact, standard medium was supplemented with 10% FCS and, since average Ca^2+^ concentration in FCS is around 14.6 mg/dL, thus 3.7 mM [47], Ca^2+^ concentration in our standard medium (10% FCS) was around 0.37 mM. When additional 2.5 mM Ca^2+^ was included, final Ca^2+^ concentration was 2.87 mM (0.37+2.5 = 2.87).

For the kinetic study, aimed to obtain growth curves (which provide indication of cell growth rate) and doubling time (DT, indicative of the proliferation rate), large and small cell lines at P5 were used. Cells were grown in four different culture conditions: 1) control (CTRL): standard medium as described above; 2) higher external calcium (2.5 mM CaCl_2_): standard medium plus 2.5 mM CaCl_2_; 3) agonist (2.5 mM+R-467): standard medium plus 2.5 mM CaCl_2_ and 3 µM NPS R-467 added for the first 24 hours; 4) antagonis/agonist (2.5 mM+2390+R-467): standard medium plus 2.5 mM CaCl_2_ and 3 µM NPS R-467, added for the initial 24 hours. In these samples, before CaSR agonist addition, cells were preincubated for 30 min with 10 µM NPS 2390. Except for the CTRL wells, CaCl_2_, NPS R-467 and NPS 2390 were added 72 hours after culture starting (cells seeding time). After 24 hours of incubation with tested compounds, culture medium was replaced with standard expansion medium in CTRL wells while medium with additional 2.5 mM CaCl_2_ in all other culture conditions. Cells of each well were detached by using 0.05% trypsin/0.02% EDTA in PBS and were counted, by dilution (1∶1) in Trypan blue, with Burker's chamber.

For growth curves, the large and small cells were seeded in all six wells of four culture plates (one for each culture condition) and cell counts were performed in six different days, every two to three days. Cells were counted at P5, on days: 1^st^, 4^th^, 7^th^, 9^th^, 11^th^, 14^th^ after treatments. The Student's *t*-test was used to evaluate statistical significances and values with P<0.05 were considered as statistically different.

For DT calculation, cells were seeded, at the density of 1000 cell/cm_2_, in the first well of a six well plate, counted every 48 hours and re-seeded in the next well of the same plate at the same density (1000 cell/cm_2_) to the end of the treatment. The DT data were calculated by using the following formula: CD  = ln(N_f_/N_i_)/ln 2 and DT = CT/CD, where DT is the cell-doubling time, CD is the cell-doubling number, and CT is the cell culture time. The proliferation rate was calculated from each passage, where N_f_ is the final number of cells and N_i_ the initial number of cells. For DT calculation, cells were counted from P5 to P8.

#### Experiment 2a: Effects of high external calcium concentration and the calcimimetic NPS R-467 on CaSR transcript relative abundance in equine UCM-MSCs

CaSR expression was evaluated on both large and small cell lines on day 4^th^ after treatment. At mRNA level, the study was performed by comparative Real Time PCR. The amplification of HPRT1 mRNA allowed us to assess the suitability of the used total mRNA extraction method and the presence of total mRNA as an endogenous control. Ct values of HPRT1 were used to normalize the expression levels of the target gene in all examined samples in both cell lines. Ovarian mural granulosa cells were used as positive controls, as CaSR expression was previously demonstrated in these cells [59]. The Student's *t*-test was used to evaluate the statistical significance of the results. Values with P<0.05 were considered as statistically different.

#### Experiment 2b: Effects of high external calcium concentration and the calcimimetic NPS R-467 on CaSR protein expression and subcellular localization in equine UCM-MSCs

At protein level, CaSR expression and subcellular localization were evaluated by immunofluorescence and confocal microscopy. Groups of 40 to 44 cells were analyzed for each experimental condition. The statistical significance of the results expressed as rates of cells showing prevailing cytoplasmic or cortical CaSR labeling was evaluated by the Chi-square test with the Yates correction for continuity. Proportion of cells showing prevailing cytoplasmic or pericortical CaSR labeling were compared between each treatment group and controls. Values with P<0.05 were considered to be significantly different.
